# Case of Pulsating Veins

**Published:** 1847-04

**Authors:** H. Marsh


*Case of Pulsating Veins.* By Sir H. Marsh, Bart.—Catherine Duffy, aged 28, was admitted into Steevens' Hospital under Sir Henry Marsh, 13th May, 1846, presenting the following appearance:

All the superficial veins of the right arm and hand are greatly dilated; those on the back of the fore-arm, above its middle, being much convoluted as well as swollen. The veins on the back of the hand are much contorted, and in various places varicose. On the little and ring fingers the veins present, in a well-marked manner, the appearance of aneurism by anastomosis, whereby these fingers are irregularly swollen to fully double their natural thickness.

A little before the axillary artery becomes brachial, or just above the lower edge of the tendon of the latissimus dorsi, the vessel becomes abruptly dilated to fully four times its natural diameter. The dilatation is of the entire circumference, and extends about two inches along the brachial artery; its calibre is tolerably uniform or cylindrical, except that on its anterior and internal surface it assumes a form somewhat irregular or nodulated. Above this dilatation, as far back as can be traced by the finger, the vessel, though not considerably dilated, feels larger than is natural, or than its corresponding portion on the opposite side. About two inches below the commencement of this dilatation, the artery as abruptly contracts. The contraction is such as to convey the idea of a cord having been tied tightly around the vessel. Immediately below this contraction the vessel appears again to dilate, but not in its entire circumference ; a pouch or sack, somewhat of the size and form of a split hazel-nut, occupies its anterior and internal surface. Between this sack and the vessel there is an obvious communication, the former being readily emptied by pressure. Below this last described tumour, till pretty near its bifurcation, the artery is of uniform calibre. It is somewhat, but inconsiderably, larger than the corresponding portion in the opposite extremity. From just above the internal condyle to its bifurcation, the the vessel is again uniformly dilated to fully double its natural dimensions. The radial and ulnar arteries are distended to pretty nearly twice their natural size, uniformly throughout their entire extent.

On the posterior and internal portion of the fore arm, corresponding to about the middle of the ulna, is a soft, compressible tumour, slightly pulsating, and in size somewhat about that of half an ordinary sized walnut. In the palm of the hand, immediately beneath the pisiform bone, is an ill-defined pulsating swelling, which resembles an aneurism by anastomosis ; beneath this, and corresponding to the cleft between the middle and index finger, is a tumour of a similar nature, somewhat less in size, and less distinctly pulsating.

Pulsation is visible immediately above the sternum, along the brachial, the radial, and the ulnar arteries ; in the tumour on the back of the fore-arm, and in those in the palm of the hand. Pulsation is not visible in the carotids, nor in the tumours on the little and ring fingers.

By pressure applied to the axillary artery all pulsation in the tumour is arrested. Fremissement is well marked along the brachial, less distinctly along the radial and ulnar arteries. On taking the patient by the hand, and using gentle pressure, a tremulous purring sensation is communicated by the tumours in the palm.

On applying the stethoscope immediately beneath the acromial end of the clavicle, there is heard an intensely loud continuous murmur. This sound becomes somewhat augmented at every ventricular systole. As the stethoscope is moved towards the sternum, the murmur becomes less and less audible. At no part of the sternum can this murmur be heard. The heart's sounds appear perfectly normal. A *bruit de soufflet* is audible along the entire course of the brachial, radial, and ulnar arteries. A murmur exactly resembling the placental soufflet is heard in the various pulsating tumours. Nothing abnormal can be recognised in any of the other vessels in the body.

The pulse at both wrists is 72, and regular ; being, however, on the affected side considerably fuller and somewhat stronger than on the other.

The temperature of the affected limb (one of the symptoms the patient complained of as most distressing), was, when measured by the thermometer, always several degrees higher than that of the other. The amount of the difference was found to vary with the condition of the circulation, being augmented by whatever produced an acceleration of the heart's action. Any slight excess of exercise, she states, causes the limb to be bathed in perspiration.

There is a dull, aching pain, of variable intensity, at all times present in the arm, from which some relief is experienced, as also a partial emptying of the vessels, and slight diminution in the size of the various tumours, by holding the arm in an elevated position.

The patient's general health does not appear to have suffered much; her strength is but little impaired, her appetite good, her bowels regular, and her sleep undisturbed. Her catamenia did not appear the last two menstrual periods, but had always been previously regular.

The patient's history is briefly as follows:

Her parents, and numerous brothers and sisters, are healthy. She has been married eight years, and has had five children, nothing remarkable as to hæmorrhage or otherwise having occurred at her confinements. Her circumstances being independent, she has never pursued a laborious life, nor employed her right arm in any way calculated particularly to fatigue it. She has ever considered herself healthy and strong, except that intestinal worms have been to her for some years a source of much trouble. She states that from childhood, so far back as she can recollect, she thinks she had observed the veins of the effected arm and hand to have been larger and more swollen than those of the other; but having experienced from this no inconvenience, she never attached to it any importance. About a year and a half ago, she, for the first time, observed a throbbing to occur in the effected arm after exertion, or increased exercise of any kind. Somewhat about the same time, as well as she can recollect, she first felt a sharp stinging pain occasionally shooting down the arm, commencing, as she describes it, at the inferior angle of the scapula, and shooting through the arm to the hand. For some time she attached but little importance to these symptoms, the pain being but occasional and momentary, and the throbbing caused alone by exertion. Since then to the present time they have been gradually becoming more distressing; the occasional sharp stinging pain has been replaced by one constant, dull, and aching; while the throbbing and distressing sense of heat, which at first was brought on alone by excess of exercise, is now ever present, even while at rest. She can give no definite information as to the period of the first appearance, or the progress of the various tumours, but is of opinion that they have all formed within the last eighteen months. She has had much medical advice, having consulted several physicians, and having been for some time in hospital previously to coming into Steevens', but is of opinion that she has derived but little benefit from treatment.

For the sake of brevity, a detail of the daily treatment has been omitted, the more especially as it appeared to exercise but little control over the disease. It consisted for the most part, of general sedatives, with the local application of refrigerating lotions, and the occasional employment of pressure,—pressure generally of the entire limb, and specially on the distinct tumours, varying its position and degree as appearance indicated. The latter part of this treatment required caution, as also a prolonged interval between the periods of its employment; for, even when inconsiderable, it soon caused no slight augmentation of the patient's sufferings. Most relief appeared to be derived from cold applications to the limb.

At the expiration of somewhat more than two months, the patient, at her own request, left hospital, having apparently derived but little benefit from its treatment.—*Dublin Quarterly Journal.*

